# Optimal Initial Blood Pressure in Intensive Care Unit Patients with Non-Traumatic Intracranial Hemorrhage

**DOI:** 10.3390/ijerph17103436

**Published:** 2020-05-14

**Authors:** Ming-Cheng Wei, Edy Kornelius, Ying-Hsiang Chou, Yi-Sun Yang, Jing-Yang Huang, Chien-Ning Huang

**Affiliations:** 1 Institute of Medicine, Chung Shan Medical University, Taichung 40201, Taiwan; fazen.tw@yahoo.com.tw (M.-C.W.); hideka@gmail.com (Y.-H.C.); 2 Department of Neurosurgery, Lee General Hospital, Yuanli Town, Miaoli 35845, Taiwan; 3 Division of Endocrinology and Metabolism, Department of Internal Medicine, Chung Shan Medical University Hospital, Taichung 40201, Taiwan; kornelius82@gmail.com (E.K.); monica119@gmail.com (Y.-S.Y.); 4 Department of Radiation Oncology, Chung Shan Medical University Hospital, Taichung 40201, Taiwan; 5 Department of Medical Imaging and Radiological Sciences, Chung Shan Medical University Hospital, Taichung 40201, Taiwan; 6 Department of Medical Research, Chung Shan Medical University Hospital, Taichung 40201, Taiwan; wchinyang@gmail.com

**Keywords:** intracranial hemorrhage, stroke, blood pressure, mortality, critical care

## Abstract

Blood pressure (BP) control is crucial for minimizing the risk of mortality and hematoma growth in patients with acute intracranial hemorrhage (ICH). We aimed to determine the optimal BP range associated with improved patient outcomes. From the Medical Information Mart for Intensive Care-III database, we identified 1493 patients (age, 18–99 years) admitted to the intensive care unit (ICU) with non-traumatic ICH. The 3-day and 14-day mortality of ICU admissions were compared at different BP ranges. Generalized additive models were used to assess the optimal range of initial mean arterial pressure, systolic blood pressure (SBP), and diastolic blood pressure, and these were identified to be 70–100, 120–150, and 60–100 mmHg, respectively. The 3-day or 14-day mortality showed U-shaped correlations with BP ranges. Our results show that an initial SBP between 120 and 150 mmHg is associated with minimal risk of mortality risk. This recommendation can assist physicians to achieve better outcomes for patients with ICH.

## 1. Introduction

Intracerebral hemorrhage (ICH) accounts for 10–15% of all strokes, and hemorrhagic strokes have a worse prognosis than ischemic strokes [1]. Evidence-based guidelines for treatment options in the acute phase of ICH [2] allow physicians to achieve the best prognosis for patients. The most important concern for clinicians is the recovery of neurological function and reduction in mortality rates by disposal caused by treatment.

In the early stages, ICH scores are used to predict mortality in patients with hemorrhagic stroke. If the ICH score is >3, the expected 30-day mortality rate is >70%. Prognostic factors include Glasgow Coma Scale (GCS) score at admission, age, bleeding volume, subtentorial hemorrhage, and intraventricular hemorrhage [3]. In addition, in the early stages of ICH, high blood pressure (BP) can cause acute hematoma growth, worsening of clinical status, and even death. Therefore, the hypothesis that lowering BP during the acute phase may reduce bleeding is reasonable, and the control of BP in the acute phase has become an important issue.

As for BP control in hemorrhagic stroke, the guidelines from the American Stroke Association Stroke Council [4] suggest that systolic blood pressure (SBP) should be reduced to <180 mmHg in the acute phases of hemorrhagic stroke. In one previous report, the absolute effect of intensive BP therapy on hematoma growth was small and the absolute difference in post-treatment volume in the intensive BP group and the control group was not significant (1.7 mL) (*p* = 0.13) [5].

New predictors of hematoma growth include initial bleeding volume greater than 16 mL (hematoma volume [HV] > 16 mL), hematoma heterogeneity (HH), and initial SBP > 160 mmHg (1.5H-SBP > 160 mmHg) [6]. Since the role of intensive reduction of high BP has not been clarified, and because BP has a limited effect on hematoma growth, this study investigated acute high BP after stroke from a different point of view.

Using the Medical Information Mart for Intensive Care (MIMIC)-III [7] database for analysis, we investigated whether initial BPs (mean arterial pressure (MAP), SBP, and diastolic blood pressure (DBP)) could predict the short-term (3-day) and medium-term (14-day) mortality after ICU admission. We aimed to determine the optimal initial range of BPs that are indicative of a good prognosis for patients with non-traumatic ICH.

## 2. Materials and Methods

### 2.1. Data Source

The MIMIC-III database was used in this study [7]. MIMIC-III investigates critical variables for improvements in clinical decision-making and epidemiological studies. The dataset contains demographic information, diagnoses of diseases, measures of vital signs, laboratory test results, medications, imaging reports, and mortality rates. The details of the MIMIC-III database can be found at http://www.nature.com/articles/sdata201635. It is a free database, and researchers requesting access to MIMIC-III must complete a Collaborative Institutional Training Initiative course on the website. Version 1.4 of MIMIC-3 includes data from 58,931 intensive care unit (ICU) stays from June 2001 to October 2012. In order to protect personal privacy, the demographics and time-related variables are encrypted.

Data availability: Data available in MIMIC-III. Johnson, A.E.W. et al. MIMIC-III, a freely accessible critical care database. Sci. Data. 3. 160035 (2016).

The Institutional Review Boards of the Beth Israel Deaconess Medical Center and Massachusetts Institute of Technology have approved the use of the MIMIC III database by any researcher who meets the data user requirements. The requirement for informed consent of the study participants was waived by approval from the relevant ethical committees. All studies were performed in compliance with the Declaration of Helsinki, which describes the ethical principles for medical research involving human subjects.

### 2.2. Study Population

**Figure 1:**
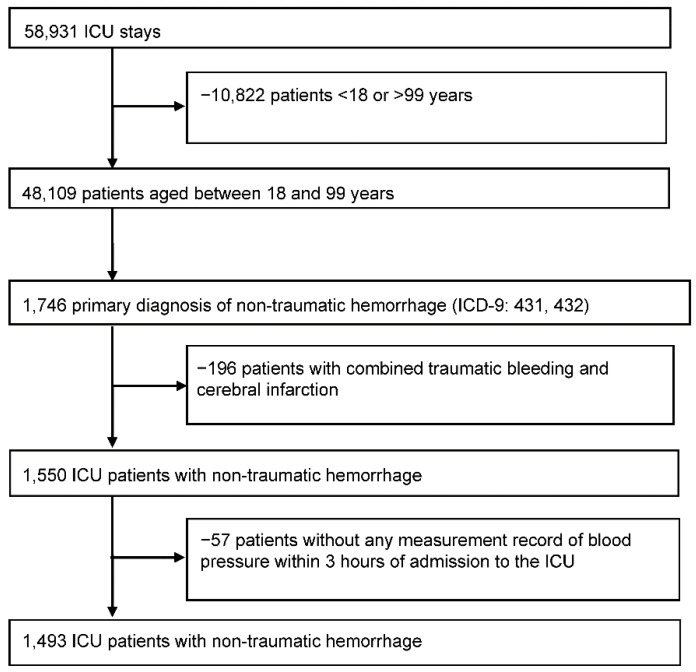
Screening of patients for ICU stays with a record of intracerebral hemorrhage (ICH) analysis. ICD-9-CM, International Classification of Diseases, 9th Revision, Clinical Modification; ICU, intensive care unit; ICH, intracerebral hemorrhage.

We identified 48,109 ICU stays in patients aged between 18 and 99 years. Initially, patients with ICH were identified by the International Classification of Diseases, 9th Revision, Clinical Modification (ICD-9-CM) codes 431 and 432 [7]. There were 1746 ICU stays with a record of ICH. Data was excluded when an ICU stay was combined with a non-traumatic intracranial hemorrhage (ICD-9-CM codes 852 and 853) or cerebral infarction (ICD-9-CM codes 433 and 434) (*n* = 196) or when there was no BP measurement within 3 h of admission to the ICU (*n* = 57). Therefore, the final dataset for analysis included 1493 ICU stays with a record of ICH (Figure 1).

### 2.3. Blood Pressure Data

BP values were identified from a chart events table that contained all primary repositories of a patient’s information obtained during their ICU stay. The electronic chart displays the patient’s routine vital signs and any additional information relevant to their care, such as ventilator settings, laboratory values, code status, mental status, etc. Item identifiers (ITEMIDs) were used to identify MAP (ITEMIDs: 456,52,6702,443,220052,220181,225312), SBP (ITEMIDs: 51,442,455,6701,220179,220050), and DBP (ITEMIDs: 8368,8440,8441,8555,220180,220051). Occasionally, the values were not manually validated by a clinical staff member, and outlier values (MAP > 300 mmHg, SBP > 400 mmHg, or DBP > 300 mmHg), or ERROR values were regarded as missing values.

We calculated the mean of MAP, SBP, and DBP values within 3 h after the patient was transferred to the ICU. First, we used generalized additive models [8] to explore the relationship between initial MAP, SBP, and DBP with 14-day mortality. We found U-shaped relationships between BPs and 14-day mortalities (Figure S1). Therefore, we classified the potential categories of blood pressure according to the results of smoothing components. MBP was divided into 3 levels (<70, 70–100, ≥100 mmHg), SBP into 4 levels (<120, 120–150, 150–170, ≥170 mmHg), and DBP into 3 levels (<60, 60–100, ≥100 mmHg).

### 2.4. Death Identification

The admissions table included DEATHTIME (time of death), which was only present if the patient died in the hospital and was almost always the same as the patients’ DISCHTIME (time of discharge). However, there may have been some discrepancies due to typographical errors. We calculated the interval between the date of transfer to the ICU and the date of death. Accordingly, we classified patients into survivors and deceased patients within 3 or 14 days after ICU admission. The 3-day and 14-day mortality was defined as short-term and medium-term mortality, respectively.

### 2.5. Covariates

We also identified the age at admission (years), sex, marital status, ethnicity, admission type, entry to the ICU, diabetes (ICD-9-CM code: 250), sepsis, and Sequential Organ Failure Assessment (SOFA) score from datasets. Furthermore, the minimum GCS score was calculated and used in the sub-group analysis. Surgery for ICH was defined by the ICD-9 procedure codes 01.18, 01.21, 01.23, 01.24, 01.25, 01.31, 01.39, 02.01, and 02.39.

### 2.6. Statistical Analysis

The number (proportion) of case mortalities were noted within 3 and 14 days of ICU admission among different MAP, SBP, and DBP groups. Multiple logistic regression was used to estimate the adjusted odds ratio (aOR) and its 95% confidence interval, when controlling for covariates (including age, sex, care unit, sepsis, and SOFA score) in the model. A Kaplan–Meier analysis was performed to estimate the cumulative survival rate across the groups, and the log-rank test was used to analyze the statistical significance. The sub-group analysis was performed for specific GCS scores and surgery groups. SAS (version 9.4; SAS Institute, Cary, NC, USA) software was used to perform the statistical analysis. A *p*-value of less than 0.05 indicated statistical significance.

## 3. Results

Table 1 shows the demographic characteristics of ICU patients with non-traumatic ICH. The proportion of men and women were 42.5% and 57.5% of the patients, respectively, and 85.6% of the patients were over 50 years of age. Overall, 20% of the patients had diabetes, 48.6% had a GCS score < 8, and 20.3% underwent surgery. Of the included patients, 204 (13.66%) and 369 (24.72%) died within 3 and 14 days, respectively.

Table 2 shows the proportion (%) and crude odds ratio of 3-day and 14-day mortality of patients with non-traumatic ICH with different levels of initial MAP, SBP, and DBP. There were relatively low risks of short or medium-term mortality when initial MAP ranged between 70 and 100 mmHg, SBP between 120 and 150 mmHg, and DBP between 60 and 100 mmHg. Compared to those with MAP between 70 and 100 mmHg, patients with MAP < 70 mmHg had significantly increased odds for 3-day and 14-day mortality. For SBP, compared with values between 120 and 150 mmHg, patients with SBP < 120 or ≥170 mmHg presented significantly higher odds of 14-day mortality, as was observed in patients who had died within 14 days. For DBP, significantly increased risk of mortality was observed in those with DBP < 60 mmHg had the.

**Figure 2:**
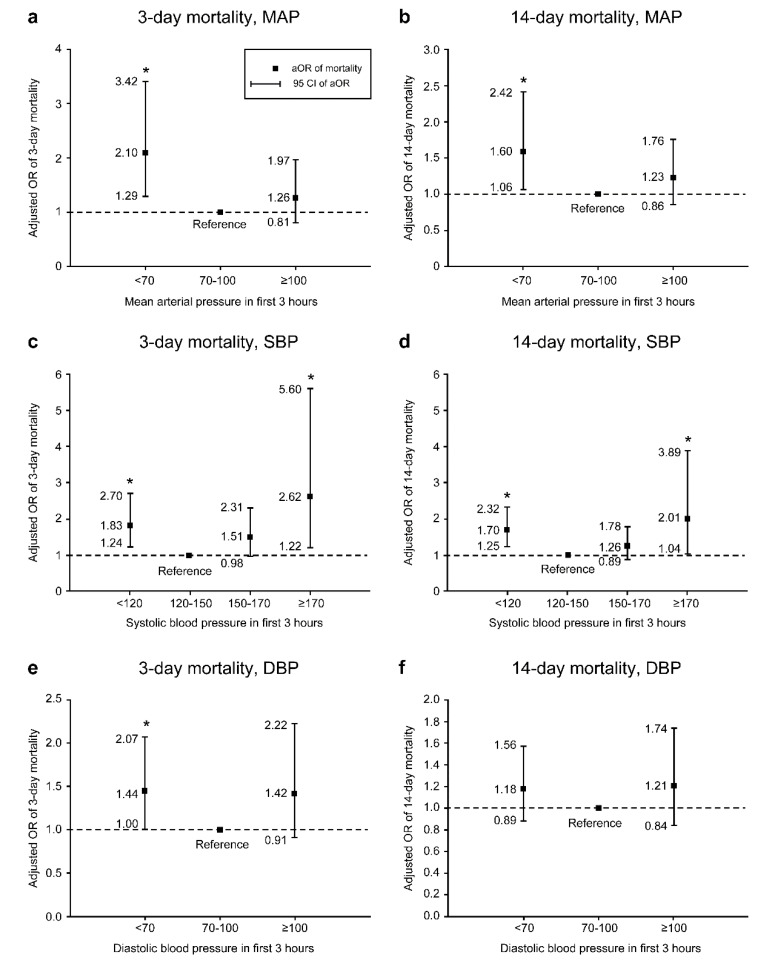
The adjusted odds ratio (aOR) of mortality in patients with non-traumatic hemorrhage (*n* = 1493). (**a**) aOR of 3-day mortality in MAP levels, (**b**) aOR of 14-day mortality in MAP levels, (**c**) aOR of 3-day mortality in SBP levels, (**d**) aOR of 14-day mortality in SBP levels, (**e**) aOR of 3-day mortality in DBP levels, (**f**) aOR of 14-day mortality in DBP levels. aORs were adjusted for sex, care-unit, age, sepsis, and Sequential Organ Failure Assessment score. MAP, mean arterial pressure; SBP, systolic blood pressure; DBP, diastolic blood pressure; OR, odds ratio; CI, confidence interval; *, *p* < 0.05.

Figure 2a–f show the aOR of 3-day and 14-day mortality in different levels of MAP, SBP, and DBP, after adjusting for sex, care-unit, age, sepsis, and SOFA score. We found a U-shaped association of BPs with 3-day and 14-day mortality rates. For initial MAP, MAP < 70 mmHg significantly increased the odds of 14-day mortality (aOR = 1.60, 95% confidence interval (CI) = 1.06 to 2.42). For initial SBP, both SBP <120 mmHg (aOR = 1.70, 95% CI = 1.25 to 2.32) and ≥170 mmHg (aOR = 2.01, 95% CI = 1.04 to 3.89) had significantly higher aOR for 14-day mortality.

Table 3 indicates the age, sex and GCS score stratified analysis. For MAP levels, the age, sex and GCS score did not significantly modify the relationship between MAP and 14-day mortality. However, we found that younger patients (18–64 years), men, and those with a high GCS score (9–15) had a negative linear (but not U-shaped association) relationship between MAP and 14-day mortality rate. For SBP levels, there was no significant interaction of age, sex, and GCS score on the effect of SBP; however, we found that an initial higher SBP (>150 mmHg) does not increase the 14-day mortality in younger patients (aged 18–64 years). For DBP levels, we found an interaction effect between sex and DBP on 14-day mortality. Higher DBP increased the mortality in women, whereas lower DBP increased the mortality in men.

**Figure 3:**
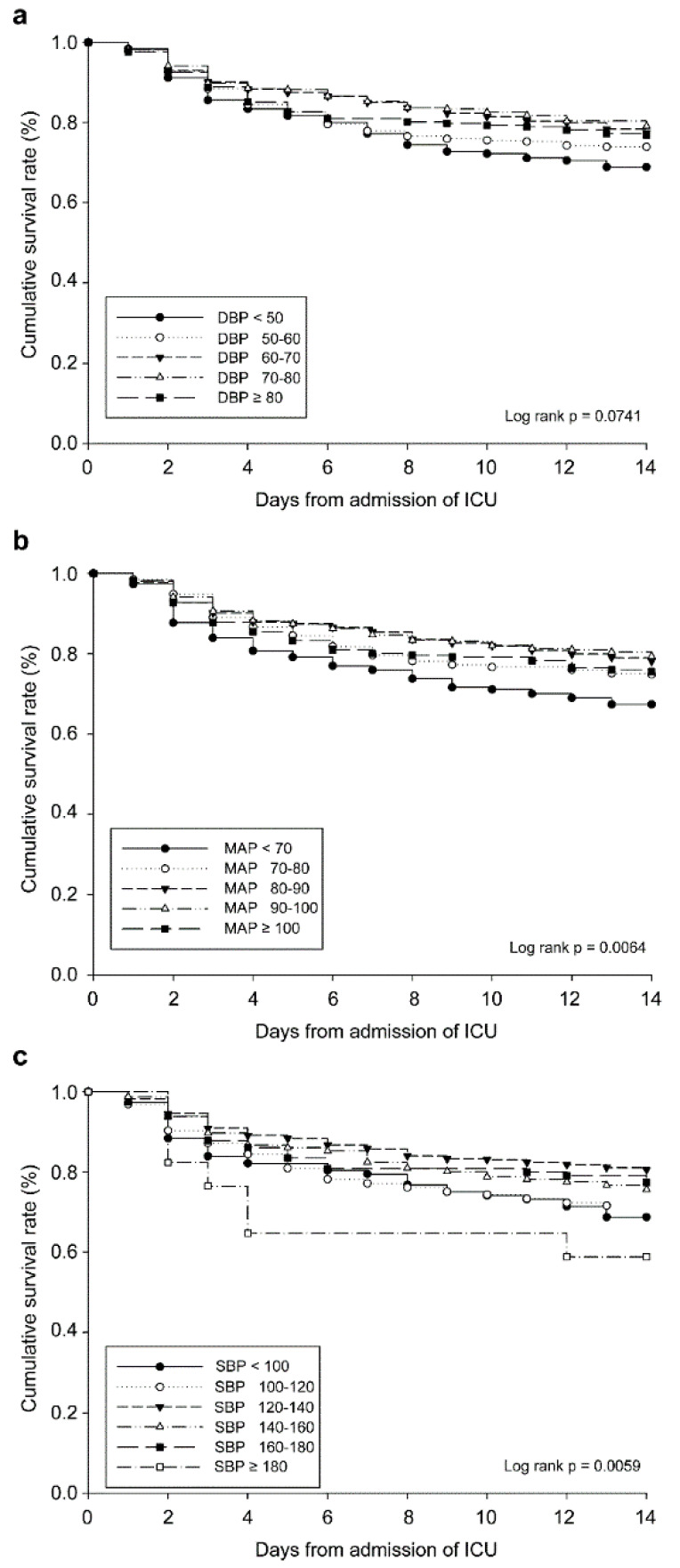
Kaplan–Meier comparison of mortality in patients with non-traumatic hemorrhage. (**a**) Mean arterial pressure. (**b**) Systolic blood pressure. (**c**) Diastolic blood pressure. Data were adjusted for sex, marital status, ethnicity, admission type, insurance type, care-unit, age, creatinine clearance level, diabetes mellitus, sepsis, and Sequential Organ Failure Assessment score. GCS, Glasgow Coma Scale.

After the data were adjusted for sex, marital status, ethnicity, admission type, insurance type, care-unit, age, creatinine clearance level, diabetes mellitus, sepsis, and SOFA score, we performed Kaplan–Meier analysis of mortality rates in patients with non-traumatic hemorrhage (Figure 3).

## 4. Discussion

The Intensive Blood Pressure Reduction In Acute Cerebral Hemorrhage Trial (INTERACT) [5,9] and INTERACT-2 [10,11] studies, designed in Australia, aimed to assess a new method to more intensively reduce early high BP. However, the study results showed no significant outcomes. Furthermore, the Antihypertensive Treatment of Acute Cerebral Hemorrhage (ATACH) [12,13] and ATACH-II [14] trials, designed in North America, which aimed to assess escalating levels of antihypertensive treatment, also failed to show significant results. A study by Alrahbi and colleagues showed that rapid reduction in BP to 110–139 mmHg did not lower the mortality or disability rate when compared to a BP reduction to 140–179 mmHg [15]. The Perioperative Antihypertensive Treatment in Patients with Spontaneous Intracerebral Hemorrhage (PATICH) study, conducted in mainland China, was designed to enhance perioperative anti-hypertensive therapy to achieve a target SBP of 120–140 mmHg in the test group, and a target SBP of 140–180 mmHg in the conservative group [16]. Intensive perioperative BP reduction 7 days postoperatively was not associated with a reduced rate of rebleeding, mortality, or other serious adverse events [16]. Therefore, rapid lowering of BP in the acute phase does not appear to decrease long-term mortality, and the association between hypertension and hematoma growth in acute cerebral hemorrhage cases remains poorly clarified.

According to our clinical observations, it can be difficult to obtain precise information about initial BP in clinical practice. Initial BP measurements may be taken in the ambulance, in the emergency room, during transfer to the ICU, or in the ICU postoperatively. BP measurements taken at each of these time points will vary because of various reasons, including movement of the patient and administration of medication. Furthermore, the initial BP measurement may have been discarded.

Therefore, to meet the clinical feasibility requirements, regardless of whether resuscitation or surgery was performed, we used BP measurements taken within 3 h of reaching the ICU as the standard initial BP measurement. The average values for each measurement were as follows: average MAP, between 70 and 100 mmHg; average SBP, between 120 and 150 mmHg; and average DBP, between 60 and 100 mmHg.

Previous results suggest that clinicians need to control SBP < 180 mmHg in patients with acute high BP after hemorrhagic stroke [4]. Furthermore, to reduce rebleeding and hematoma growth, it is feasible to further control SBP to <160 mmHg [6]; however, it is advised that clinicians pay attention to increased risk of renal function damage caused by rapid BP reduction (9.0% vs. 4.0%, *p* = 0.002) [17]. Our results suggested an optimal SBP range of 120–150 mmHg for minimal risk of mortality and avoidance of hematoma growth.

Our results also showed that most deaths occurred in patients with severe disease (GCS score < 8). Consistent with previous studies, the absolute effect of intensive BP therapy on hematoma growth appeared to be minimal [5]. When we performed a subgroup analysis of mild and severe disease groups (Table 3), SBP had significant effect on severe disease (GCS score < 8) patients.

ICH rebleeding occurs frequently within 6 to 12 h of stroke; therefore, delays in the treatment of acute high BP may have negative consequences [18]. In patients with acute supratentorial ICH, SBP > 180 mmHg can independently predict hematoma growth [19].

To minimize the deleterious effects of acute high BP on hematoma growth and clinical outcomes, theoretically, intensive anti-hypertensive therapy can be used to maintain a stable BP and reduce poor outcomes. One large randomized controlled trial (RCT) demonstrated that safer BP control correlated with a better prognostic trend, and regardless of whether acute high BP occurs, BP can be significantly reduced to achieve this goal [20].

Based on the results of INTERACT-2 [10,11], the American Heart Association Guidelines suggest that BP can be safely reduced to 140 mmHg in the early stage in patients with an SBP of 150−220 mmHg without contraindications to intensive BP therapy with ICH [2]. However, a recent meta-analysis, including five large RCTs—INTERACT-1 in 2008 [5], Rapid Blood Pressure Reduction (RBPR) ICH in 2008 [21], ICH Acutely Decreasing Arterial Pressure Trial (ADAPT) in 2013 [22], INTERACT-2 in 2013 [10], and ATACH-II in 2016 [17]—indicated that elevated BP in patients with acute ICH is safe. Furthermore, it appeared that intensive anti-hypertension did not improve functional outcomes or 3-month mortality rates [23].

Therefore, there is still intense debate about whether intensive anti-hypertension should be performed in patients with acute ICH [24]. A previous report indicated that reducing SBP to 140 mmHg is safe and beneficial [2]; however, in our study, low MAP (<70 mmHg) or SBP (<120 mmHg) was related to 3-day and 14-day mortality. Moreover, high SBP (≥170 mmHg) showed the strongest relationship with 3-day and 14-day mortality (Figure 2). In addition, new predictors of hematoma enlargement include an initial bleeding volume > 16 mL (HV > 16 mL), HH, and an initial SBP > 160 mm Hg (1.5H-SBP > 160 mmHg) [6]. Based on previous reports and our results of statistical analyses, we suggest that the optimal controlled SBP range is between 120 and 150 mmHg in patients with ICH with acute high BP as this range is associated with the lowest mortality.

The main limitation of this study is that it is a retrospective cohort study, and could not provide further insight into the mechanisms underlying BP control. The intermediate influential factors could not be further analyzed, and only statistical analysis of the correlation between initial BP and mortality was performed. As is with all routinely collected datasets, only a limited range of variables were collected in the MIMIC-III database that could be included in the models. Some of these variables with significant prognostic relevance to ICH are missing from analyses such as time from symptom onset, baseline neurological scale scores (beyond use of GCS score), and BP lowering treatment before transfer of patients to NICU. However, the main strength of this study is that it was based on a large number of hospital ICU databases, with high data credibility and an expert, unbiased evaluation of the diagnoses.

Future research must focus on patients with hemorrhagic stroke, specifically aimed at gaining knowledge on how to control BP, reduce intracranial pressure, and increase perfusion pressure. These could be used to find the primary focus for treatment and how these factors influence each other. These questions need to be answered to establish an appropriate BP range and goals to reduce mortality and morbidity.

## 5. Conclusions

According to previous reports, acute hypertension in patients with hemorrhagic stroke may affect bleeding and worsen prognosis. The question remains as to whether it is necessary to actively lower BP to below 140 mmHg in these patients. Using the large, freely available MIMIC-III database for this analysis, we found that the optimal maintenance range for SBP was between 120 and 150 mmHg, as this level conferred minimal short-term and middle-term mortality rates. Our results can safely assist physicians to achieve accurate BP control in clinical settings.

## Figures and Tables

**Table 1:** Demographic characteristics of patients with non-traumatic intracranial hemorrhage.

	Non-Traumatic Hemorrhage	Mortality Event (Row Percent)	
	*n* (Column Percent)	3-Day	14-Day
All	1493 (100%)	204 (13.66%)	369 (24.72%)
Sex			
Female	635 (42.53%)	92 (14.49%)	173 (27.24%)
Male	858 (57.47%)	112 (13.05%)	196 (22.84%)
Marital status			
Missing	109 (7.30%)	35 (32.11%)	59 (54.13%)
Unmarried	311 (20.83%)	27 (8.68%)	47 (15.11%)
Married	764 (51.17%)	98 (12.83%)	181 (23.69%)
Widowed	207 (13.86%)	27 (13.04%)	49 (23.67%)
Divorced	79 (5.29%)	8 (10.13%)	22 (27.85%)
Others	23 (1.54%)	9 (39.13%)	11 (47.83%)
Ethnicity			
White	1084 (72.61%)	140 (12.92%)	259 (23.89%)
Black	111 (7.43%)	13 (11.71%)	23 (20.72%)
Hispanic or Latino	59 (3.95%)	10 (16.95%)	15 (25.42%)
Asian	56 (3.75%)	12 (21.43%)	17 (30.36%)
Others	55 (3.68%)	6 (10.91%)	12 (21.82%)
Unknown	128 (8.57%)	23 (17.97%)	43 (33.59%)
Admission type			
Elective	35 (2.34%)	1 (2.86%)	2 (5.71%)
Emergency	1436 (96.18%)	201 (14.00%)	363 (25.28%)
Urgent	22 (1.47%)	2 (9.09%)	4 (18.18%)
Insurance			
Government	42 (2.81%)	2 (4.76%)	4 (9.52%)
Medicaid	110 (7.37%)	12 (10.91%)	22 (20.00%)
Medicare	856 (57.33%)	128 (14.95%)	249 (29.09%)
Private	463 (31.01%)	53(11.45%)	84 (18.14%)
Self-pay	22 (1.47%)	9 (40.91%)	10 (45.45%)
Care unit			
CCU	57 (3.82%)	9 (15.79%)	19 (33.33%)
CSRU	22 (1.47%)	5 (22.73%)	7 (31.82%)
MICU	298 (19.96%)	41 (13.76%)	86 (28.86%)
SICU	850 (56.93%)	104 (12.24%)	190 (22.35%)
TSICU	266 (17.82%)	45 (16.92%)	67 (25.19%)
Age at admission (years)			
18–34	47 (3.15%)	7 (14.89%)	8 (17.02%)
35–49	168 (11.25%)	18 (10.71%)	29 (17.26%)
50–64	419 (28.06%)	56 (13.37%)	87 (20.76%)
65–79	531 (35.57%)	73 (13.75%)	142 (26.74%)
80–99	328 (21.97%)	50 (15.24%)	103 (31.40%)
Diabetes			
No	1183 (79.24%)	156 (13.19%)	290 (24.51%)
Yes	310 (20.76%)	48 (15.48%)	79 (25.48%)
Explicit sepsis			
No	1423 (95.31%)	193 (13.56%)	340 (23.89%)
Yes	70 (4.69%)	11 (15.71%)	29 (41.43%)
SOFA score			
0–8	177 (11.86%)	8 (4.52%)	19 (10.73%)
8–10	645 (43.2%)	44 (6.82%)	98 (15.19%)
10–11	230 (15.41%)	31 (13.48%)	52 (22.61%)
≥11	441 (29.54%)	121 (27.44%)	200 (45.35%)
GCS score			
Missing	1 (0.07%)	1 (100.00%)	1 (100.00%)
3–8	726 (48.63%)	186 (25.62%)	310 (42.70%)
9–12	215 (14.40%)	8 (3.72%)	26 (12.09%)
13–15	551 (36.91%)	9 (1.63%)	32 (5.81%)
Surgery for ICH			
No	1190 (79.71%)	186 (15.63%)	323 (27.14%)
Yes	303 (20.29%)	18 (5.94%)	46 (15.18%)

SOFA, Sequential Organ Failure Assessment; GCS, Glasgow Coma Scale; CCU, Coronary Care Unit; CSRU, Cardiac Surgery Recovery Unit; MICU, Medical Intensive Care Unit; SICU, Surgical Intensive Care Unit; TSICU, Trauma/Surgical Intensive Care Unit; ICH, Intracerebral Hemorrhage.

**Table 2:** The 3-day and 14-day mortality rate in ICU patients with non-traumatic hemorrhage represented by their mean arterial pressure (MAP), systolic blood pressure (SBP), and diastolic blood pressure (DBP) levels.

	3-Day Mortality Rate		14-Day Mortality Rate	
	Death (%)	Crude OR (95% CI)	Death (%)	Crude OR (95% CI)
**MAP, mmHg**				
<70 (*n* = 133)	31 (23.31%)	2.15 (1.39–3.34)	51 (38.35%)	2.07 (1.42–3.02)
70–100 (*n* = 1140)	141 (12.37%)	Reference	263 (23.07%)	Reference
≥100 (*n* = 220)	32 (14.55%)	1.21 (0.80–1.83)	55 (25.00%)	1.11 (0.80–1.55)
**SBP, mmHg**				
<120 (*n* = 346)	60 (17.34%)	1.65 (1.16–2.35)	110 (31.79%)	1.72 (1.30–2.28)
120–150 (*n* = 826)	93 (11.26%)	Reference	176 (21.31%)	Reference
150–170 (*n* = 275)	39 (14.18%)	1.30 (0.87–1.95)	65 (23.64%)	1.14 (0.83–1.58)
≥170 (*n* = 46)	12 (26.09%)	2.78 (1.39–5.56)	18 (39.13%)	2.37 (1.28–4.39)
**DBP, mmHg**				
<60 (*n* = 427)	72 (16.86%)	1.54 (1.11–2.14)	127 (29.74%)	1.46 (1.12–1.91)
60–100 (n = 824)	96 (11.65%)	Reference	185 (22.45%)	Reference
≥100 (*n* = 242)	36 (14.88%)	1.33 (0.88–2.00)	57 (23.55%)	1.06 (0.76–1.49)

MAP, mean arterial pressure; SBP, systolic blood pressure; DBP, diastolic blood pressure; OR, odds ratio; CI, confidence interval.

**Table 3:** Results of stratified analysis for adjusted odds ratio of 14-day mortality in ICU patients with non-traumatic hemorrhage estimated by their MAP, SBP, and DBP levels.

	aOR (95% CI) of 14-Day Mortality after ICU Admission			
	**Levels of MAP, mmHg**			
	**<70**	**70–100**	**≥100**	
**Age subgroups**				
18–64 years	2.62 (1.25–5.51)	Reference	0.89 (0.49–1.61)	
65–99 years	1.32 (0.80–2.18)	Reference	1.46 (0.92–2.31)	
*p*_interaction_ = 0.1080				
**Sex subgroup**				
Women	1.77 (0.99–3.19)	Reference	1.92 (1.14–3.22)	
Men	1.38 (0.75–2.53)	Reference	0.80 (0.47–1.34)	
*p*_interaction_ = 0.0712				
**GCS subgroup**				
3–8	1.52 (0.89–2.59)	Reference	1.13 (0.73–1.75)	
9–15	1.43 (0.59–3.44)	Reference	0.59 (0.20–1.73)	
*p*_interaction_ = 0.2563				
	**Levels of SBP, mmHg**			
	**<120**	**120–150**	**150–170**	**≥170**
**Age subgroups**				
18–64 years	1.93 (1.16–3.22)	Reference	1.01 (0.52–1.95)	0.97 (0.26–3.61)
65–99 years	1.58 (1.06–2.37)	Reference	1.36 (0.90–2.06)	2.50 (1.16–5.43)
*p*_interaction_ = 0.7159				
**Sex subgroup**				
Women	1.84 (1.15–2.94)	Reference	1.77 (1.05–2.97)	2.29 (0.86–6.11)
Men	1.60 (1.05–2.45)	Reference	0.99 (0.61–1.60)	1.53 (0.61–3.80)
*p*_interaction_ = 0.4187				
**GCS subgroup**				
3–8	1.61 (1.08–2.40)	Reference	1.16 (0.75–1.79)	2.82 (1.20–6.65)
9–15	1.49 (0.75–2.94)	Reference	1.13 (0.51–2.46)	All survived
*p*_interaction_ = 0.8902				
	**Levels of DBP, mmHg**			
	**<60**	**60–100**	**≥100**	
**Age subgroups**				
18–64 years	1.35 (0.77–2.37)	Reference	1.01 (0.58–1.77)	
65–99 years	1.18 (0.84–1.66)	Reference	1.33 (0.81–2.20)	
*p*_interaction_ = 0.3854				
**Sex subgroup**				
Women	0.93 (0.60–1.43)	Reference	1.76 (1.01–3.08)	
Men	1.42 (0.95–2.12)	Reference	0.85 (0.51–1.39)	
*p*_interaction_ = 0.0281				
**GCS subgroup**				
3–8	1.14 (0.79–1.66)	Reference	1.06 (0.68–1.65)	
9–15	1.09 (0.59–2.02)	Reference	0.68 (0.25–1.87)	
*p*_interaction_ = 0.6010				

aOR, odds ratio of 14-day mortality adjusted for sex, care-unit, age, sepsis, and Sequential Organ Failure Assessment score. MAP, mean arterial pressure; SBP, systolic blood pressure; DBP, diastolic blood pressure; OR, odds ratio; CI, confidence interval.

## Supplementary Materials

The following are available online at https://www.mdpi.com/1660-4601/17/10/3436/s1, Figure S1: The spline transformations for MAP, SBP, and DBP to assess the odds for 14-day mortality. Smoothing components for (**a**) MBP, (**b**) SBP, and (**c**) DBP. MAP, mean arterial pressure; SBP, systolic blood pressure; DBP, diastolic blood pressure.
