# Supplementary material for: Optimal Initial Blood Pressure in Intensive Care Unit Patients with Non-Traumatic Intracranial Hemorrhage
Source: Int J Environ Res Public Health. 2020 May 14;17(10):3436. doi: 10.3390/ijerph17103436 (PMC7277579; doi:10.3390/ijerph17103436)
Supplement: Supplementary file 1 [file ijerph-17-03436-s001.pdf]

International Journal of
Environmental Research and Public Health

MDPI

Supplementary Material:

(a) Smoothing components for MBP

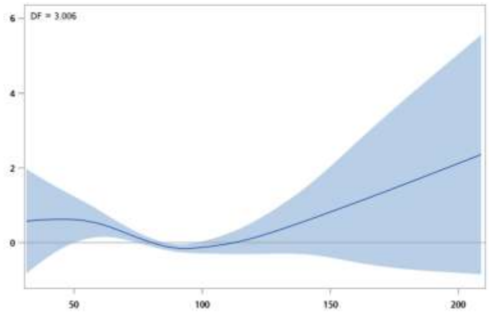

(b) Smoothing components for SBP

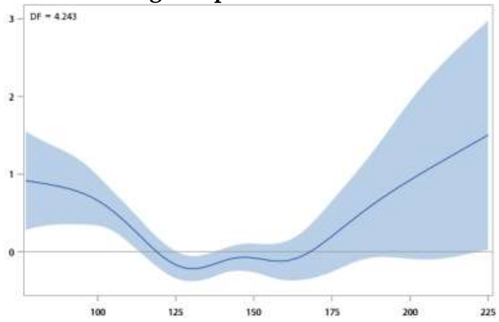

(c) Smoothing components for DBP

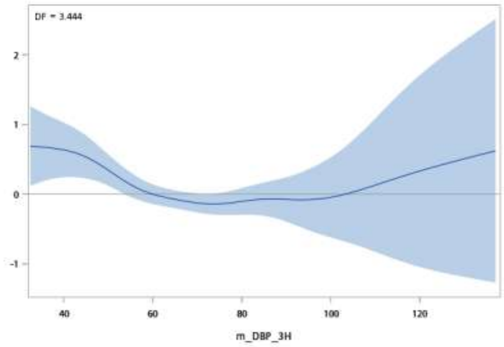

Figure S1. The spline transformations for MAP, SBP, and DBP to assess the odds for 14-day mortality. Smoothing components for (a) MBP, (b) SBP, and (c) DBP. MAP, mean arterial pressure; SBP, systolic blood pressure; DBP, diastolic blood pressure.
